# Digital Support to Multimodal Community-Based Prehabilitation: Looking for Optimization of Health Value Generation

**DOI:** 10.3389/fonc.2021.662013

**Published:** 2021-06-17

**Authors:** Anael Barberan-Garcia, Isaac Cano, Bart C. Bongers, Steffen Seyfried, Thomas Ganslandt, Florian Herrle, Graciela Martínez-Pallí

**Affiliations:** ^1^ Prehabilitation Unit, Hospital Clínic de Barcelona, Barcelona, Spain; ^2^ Instituto de Investigaciones Biomédicas August Pi i Sunyer (IDIBAPS), Barcelona, Spain; ^3^ Departemenr of Medicine, Universitat de Barcelona, Barcelona, Spain; ^4^ Department of Nutrition and Movement Sciences, School of Nutrition and Translational Research in Metabolism (NUTRIM), Maastricht University, Maastricht, Netherlands; ^5^ Department of Epidemiology, Care and Public Health Research Institute (CAPHRI), Maastricht University, Maastricht, Netherlands; ^6^ University Medical Centre Mannheim, Medical Faculty Mannheim, University of Heidelberg, Mannheim, Germany; ^7^ Anesthesiology Medicine Department, Hospital Clínic de Barcelona, Barcelona, Spain

**Keywords:** exercise training and nutrition counseling, psychological well-being, physical activity, technology – ICT, eHealth, prehabilitation, mHealth, behavioral change

## Abstract

Prehabilitation has shown its potential for most intra-cavity surgery patients on enhancing preoperative functional capacity and postoperative outcomes. However, its large-scale implementation is limited by several constrictions, such as: i) unsolved practicalities of the service workflow, ii) challenges associated to change management in collaborative care; iii) insufficient access to prehabilitation; iv) relevant percentage of program drop-outs; v) need for program personalization; and, vi) economical sustainability. Transferability of prehabilitation programs from the hospital setting to the community would potentially provide a new scenario with greater accessibility, as well as offer an opportunity to effectively address the aforementioned issues and, thus, optimize healthcare value generation. A core aspect to take into account for an optimal management of prehabilitation programs is to use proper technological tools enabling: i) customizable and interoperable integrated care pathways facilitating personalization of the service and effective engagement among stakeholders; ii) remote monitoring (i.e. physical activity, physiological signs and patient-reported outcomes and experience measures) to support patient adherence to the program and empowerment for self-management; and, iii) use of health risk assessment supporting decision making for personalized service selection. The current manuscript details a proposal to bring digital innovation to community-based prehabilitation programs. Moreover, this approach has the potential to be adopted by programs supporting long-term management of cancer patients, chronic patients and prevention of multimorbidity in subjects at risk.

## Introduction

Prehabilitation can be defined as a preventive intervention including patient-tailored therapies encompassing optimization of underlying chronic medical conditions, promotion of physical activity and nutritional and psychological support. Prehabilitation programs are designed to optimize the physical and psychological condition of patients undergoing major elective surgery with the final aim to improve clinical outcomes and foster post-surgical functional recovery. The intervention has shown its potential for healthcare value generation in different randomized controlled trials (1–5). However, despite international experts’ endorsements (6–10), its implementation as a standard of care within the Enhanced Recovery After Surgery (ERAS) recommendations (11) is still pending.

Limitations of the current evidence on effectiveness of prehabilitation are the heterogeneity among the studies. The patient population enrolled varies greatly, and it is unclear whether all patients benefit or whether only those deemed at higher risk for surgery benefit. Characterization of responders to preoperative exercise training has not been investigated thoroughly and the variety of outcome measures that exist in current literature make comparisons between studies difficult. Despite high intensity exercise training has proven effective (1–3), the type and intensity of exercise training that provides best outcomes is still a controversial hot topic (12). To overcome these well-identified aspects limiting adoption of prehabilitation before major surgery as a routine practice in different healthcare settings, multicenter, international trials with adequate sample size and appropriate power are required.

It is of note, however, that consolidated results of the ongoing PAPRIKA project (2019–21) in Barcelona (13) have identified five actionable areas that seem to play a pivotal role to ensure successful scale-up and sustainability of prehabilitation in the clinical setting. The project clearly indicates the need for: i) Refining the characteristics of the intervention; ii) Building capacity and enhancing service delivery; iii) Risk assessment and personalization; iv) Mature digital support; and, v) Transfer of the service to the community, preserving high-intensity exercise training.

Within this scenario, we believe that digital innovation can facilitate large-scale deployment of successful community-based personalized prehabilitation programs (14–16) by supporting: i) deep remodeling of case management strategies fostering an effective communication and engagement among healthcare professionals, as well as between healthcare professionals and patients and caregivers; ii) effective behavioral change techniques fostering self-efficacy and adherence to community-based interventions (i.e. remote monitoring, goal setting, feedback and educational material, among others); and, iii) decision support system tools for enhanced risk assessment and personalized service selection.

The current manuscript details a proposal to bring digital innovation to novel community-based prehabilitation programs, with special focus on its applicability. The introduction of the Health-Circuit approach will facilitate a “connected experience” for both the patient and the healthcare professionals fostering engagement into the care management process. Moreover, this proposal has the potential to be adopted by programs supporting long-term management of cancer patients (17) and chronic patients, as well as prevention of multimorbidity in subjects at risk. The final milestone would be the optimization of long-term self-management programs with proven health value generation.

## Digital Innovation Enabling Community-Based Prehabilitation

A core aspect to take into account for an optimal management of prehabilitation programs is to foster digital innovation to effectively enable: i) change of management paradigm to support collaborative case management; ii) effective engagement between stakeholders by customizable and interoperable tools providing communication and information sharing between all stakeholders to avoid fragmentation of care; iii) compliance with data security and privacy regulations; iv) customizable and interoperable integrated care pathways facilitating personalization of the service and effective engagement among stakeholders; v) remote monitoring (i.e. physical activity, physiological signs and patient-reported outcomes and experience measures, among other aspects) to support patient adherence to the program, empowerment for self-management and promotion of healthy lifestyles; and, vi) the use of health risk assessment tools supporting decision making, preventive medicine and monitoring of key performance indicators.

It is important to highlight that continuous and precise telemonitoring of patients under the umbrella of a prehabilitation program, merged with traditional perioperative assessment variables (i.e. American Society of Anesthesiologists risk score (18), GLIM criteria for the diagnosis of malnutrition (19), pre-albumin), is a promising source of comprehensive information to be analyzed in an integrative manner by computational models in order to enhance surgical risk assessment and stratification to potentially characterize responders and inform personalized service selection. The digital innovation to community-based prehabilitation programs presented in the current manuscript, and currently being developed at Hospital Clínic de Barcelona (HCB), proposes the use of smart and adaptive case management (20) tools shaping a common digital ecosystem among stakeholders without requiring tight integration with existing electronic medical records. This adopted health-system approach allows better coordination among specialized teams (i.e. surgery, anesthesiology, oncology, physical therapy) within the hospital, as well as vertical (with primary care) and horizontal integrations (i.e. primary care, health clubs, sport centers) to constitute a functional prehabilitation unit that proactively establishes co-designed work plans, trusted conversations, and exchanges relevant case data. Therefore, the digital support aims to tighten engagement of professionals with care coordination activities optimizing both value and costs (i.e. LEAN approach) (21) promoting an active role of patients thanks to an artificial intelligence-supported, cloud-based, and general data protection regulation (GDPR)-compliant communication channel (professionals’ backend). Moreover, it also includes a mobile app to allow the prehabilitation team to communicate among them and with patients, which results in higher service effectiveness and fewer unplanned events.

**Figure 1:**
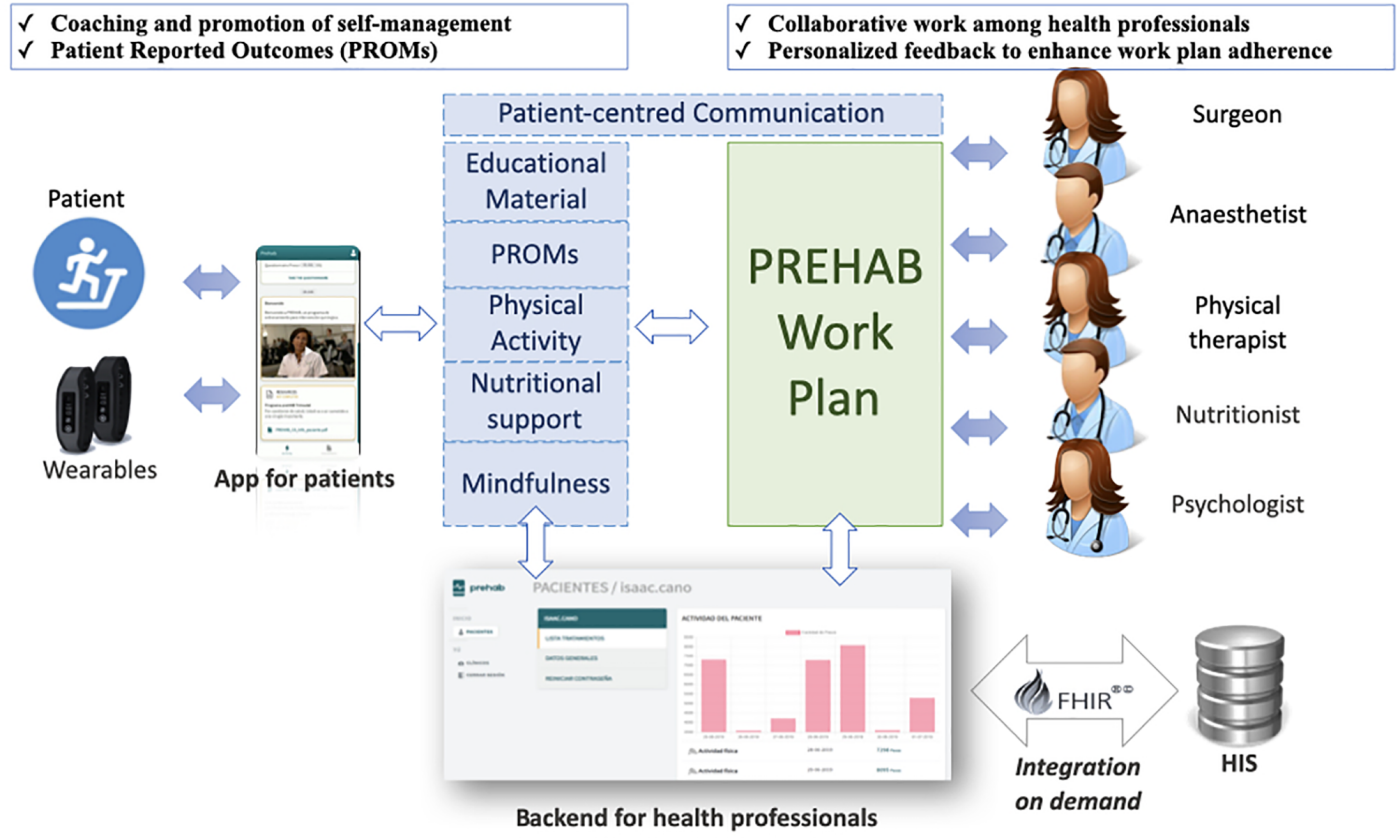
General description of the Health-Circuit approach adopted by the integrated set of smart and dynamic case management tools (PREHAB) of the EIT-Health supported innovation action PAPRIKA (2019–21). FHIR, Fast Healthcare Interoperability Resources; HIS, Hospital Information System.

The aforementioned approach to community-based prehabilitation programs leverages the Catalan best practice in digitally-enabled person-centered care (22) and the results of the EIT-Health supported innovation project PAPRIKA (2019–21) (13). PAPRIKA offers a prototyped and piloted digital health platform, co-designed by healthcare professionals along with prehabilitation patients and caregivers. Main functionalities of the digital health platform are summarized in **Figure 1** and are also discussed below.

### Functionalities of the Professionals’ Backend

The professionals’ backend allows the prehabilitation team members to prescribe and monitor the tasks status for patients’ self-management, including: i) advices for enhanced management of multimorbidity; ii) physical activity goals; iii) nutritional advices; iv) mindfulness exercises in audio format; v) consulting images for the nutritional diary; and, vi) predefined data collection instruments (i.e. hospital anxiety and depression scale, Borg scale). Moreover, prehabilitation professionals have access to a multimedia chat to communicate with patients, which is planned to evolve towards a patient-centered communication channel for remote patient consultation (tele- or videoconferencing) and remote teamwork among professionals (i.e. clinical case discussion, team coordination).

In terms of adaptive case management features, the professionals’ backend currently supports a work-flow engine that allows the creation from scratch and edition of prehabilitation work-plans, which can be customized with specific data collection instruments and periodic notifications to facilitate patient engagement. It is important to highlight that the professionals’ backend can use a HL7-FHIR middleware for standard-based integration with site-specific electronic medical records (i.e. SAP®) and electronic case report form (eCRF) for real-world cohorts [i.e. REDCap® (23)]. However, it is designed to operate on top of existing health information systems, without tight integration requirements. Health Information Exchange is expected to take place within patient-centered conversations when considered necessary by healthcare professionals.

### Functionalities of the Mobile App

The mobile app provides patients access to a follow-up timeline to check-out their daily/weekly evolution of prehabilitation goals and achievements. Moreover, Bluetooth connectivity with physical activity trackers facilitates the follow-up of physical activity goals.

In terms of communication, patients have a bidirectional messaging functionality supporting both text and images. Moreover, patients can also access predefined educational material in portable document format (PDF) and video formats and answer predefined data collection instruments to report their outcomes and/or experiences. The mobile app for patients is also planned to evolve toward a patient-centered communication channel for remote patient consultation (tele- or videoconferencing).

## Main Challenges for Digital Innovation in Surgical Prehabilitation

Compared to other sectors, healthcare (and in turn in surgical prehabilitation) has traditionally been slower in adopting digitalization. Based on the outcome of a recent state-of-research analysis (24) three potential answers could be found as to why the healthcare industry is lagging behind other sectors in its digital transformation. Firstly, researchers refer to concerns around data security that lead to patients’ rejection, as well as regulatory barriers for data use. Second, although health risk assessment is considered to enhance personalized and predictive medicine, its design and implementation is linked to complex processes that require specific expertise in data analytics. Third, healthcare professionals partly hinder further patient empowerment, mainly because of operational changes required to manage novel-patient centered value-based interventions. However, the ongoing COVID-19 pandemic is demonstrating the urgent necessity for the digital transition in the healthcare system (25–27).

In the prehabilitation arena (28, 29), digitalization should facilitate optimization of the service as well as its transference to the community, with special emphasis on a value-based and patient-centered approach appealing for innovative financing solutions. To this end, the Health-Circuit approach described above has three favorable traits. Firstly, building on top of existing health information systems, without requiring tight systems integration, it solves lack of health information exchange generated by health information silos, which often creates frustration among health professionals. A second aspect is that it solved the communication problem with flexibility for the care team: healthcare professionals, patients/careers and the community (e.g. Health clubs, wellness centres, etc.), which should facilitate its use in highly heterogeneous scenarios. Last but not least, this setting acknowledges the key role of co-design, flexibility, and customization as a basic pillar to minimize a professional’s resistance to operational changes.

The approach shows high potential for transferability and can lead to enhancement of current strategies for the management of patients with complex chronic conditions, even beyond the perioperative care period. Prehabilitation is raising increasing interest in non-surgical areas like in oncological patients to increase of both functional capacity and resilience before, during and after treatment (30), as well as in frail elderly individuals for prevention of falls. This indicates a potentially high relevance of the approach adopted in Barcelona at both healthcare and societal levels, beyond the specific preoperative focus. Moreover, characteristics of the digital support inherently ensure transferability of methods, digital health tools, and outcomes due to alignment with activities of relevant international societies (i.e. provide an example).

## Community-Based Prehabilitation Programs: Physical Activity as a Use Case

Surgical prehabilitation can be defined as a multicomponent that includes personalized preventive interventions aiming to improve a patient’s health status to enhance perioperative outcomes. In that sense, it can be conceptualized as a multimodal program to be tailored to each patient’s modificable risk factors, in terms of: i) optimization of multimorbidity management; ii) type of modules included (i.e. exercise training, physical activity, nutritional optimization, behavioral cognitive techniques, alcohol and smoking cessation, hemoglobin optimization); iii) total volume of each module taking into account: frequency of treatment administration (i.e. days per week), intensity of each treatment session, time of each single session of treatment administration (i.e. minutes) and total duration of each treatment module (i.e. days or weeks); and, iv) degree and frequency of monitoring the response of each module. As such, the total volume and context for administering each module will be directly connected to a patient’s needs, while frequently monitoring the dose-response relationship. As stated in the heading of this section, the current focus is the physical activity component of prehabilitation programs as a use case to exemplify prescription of its volume, monitoring and modularization of the service.

### Physical Activity Prescription in a Digital Scenario

There is strong evidence that lower levels of physical activity are related to poor health outcomes (31). Moreover, reduced physical activity increases the possibilities of developing most prevalent chronic conditions (32–37), including cancer (38). Furthermore, a growing body of evidence suggests positive effects of physical exercise on cancer specific as well as all-cause mortality (39, 40). Physical activity is defined as any bodily movement produced by skeletal muscles resulting in energy expenditure, while physical exercise is a subset of physical activity that is planned, structured, repetitive and purposeful. Activities of daily living are another subset of physical activity and this term refers to a set of basic, everyday tasks required for personal self-care and independent living (41). Finally, physical inactivity is a term commonly used to designate a level of physical activity that is below a specified threshold.

As a lower preoperative aerobic capacity is independently associated with worse postoperative outcomes in major abdominal surgery, we are interested in applying the above mentioned concepts within a prehabilitation program by stimulating both daily physical activity and physical exercise training. As such, the aim is to optimally increase preoperative aerobic capacity, specifically focused at those patients with a low aerobic capacity, in order to improve postoperative patient- and treatment-related outcomes. In that sense, each modality requires a specific type of setting, and promotion, assessment, and evaluation methods and devices as discussed below.

Although the prehabilitation model proposed in the current article is mainly based in the community setting, we consider the realization of a participative group sessions with a behavioral cognitive therapy approach in order to: i) educate on physical activity and physical exercise training performance (i.e. solving doubts, identifying “false myths” on the topic, alarm signs during exercise); ii) co-design the intervention while taking barriers and facilitators into account; iii) enhance a patient’s self-efficacy and motivation and commitment with the work-plan; and, iv) educate on the use of the digital solutions supporting the intervention.

In terms of physical activity monitoring, we can divide the existing portable devices in two main groups, namely, pedometers and accelerometers. Firstly, pedometers are devices which measure the number of steps performed in a given period of time and have proven a positive role as a motivational tool to increase physical activity levels (42). On the other hand, accelerometers are portable devices that detect acceleration, thereby reflecting bodily movement that may provide an estimate of time spent above or below a pre-determined physical activity threshold. However, due to higher costs and difficulty with data analysis and management the use of accelerometers is typically limited to research. In contrast, pedometers are more user-friendly, cheaper and, thus, more likely to be adopted for clinical and real-world applications. In the prehabilitation field, a pedometer-based physical activity plan seems as an interesting module to include in multimodal prehabilitation programs (3, 4, 43, 44), especially to complement high-intensity exercise training modules. In terms of tailoring pedometer-based programs, there are well-established values that can be used as a theoretical framework to personalize the amount of steps/day to each type of patient included in prehabilitation (45, 46).

In the community-based physical exercise training scenario, frequently used and accessible tools for monitoring exercise training intensity can be divided in two main groups: heart rate monitors and self-perceived exertion level scales (47). Both tools can be implemented into mobile digital solutions and are also easily managed by patients. Moreover, most of physical activity and heart rate monitoring devices, already available in the market, provide interesting and user-friendly app and web-based interfaces. These interfaces can provide information on patient’s work-plan adherence regarding predefined goals, including for example adherence to physical exercise training, symptoms experienced during physical activity, levels of stress (visual analog scales), and daily caloric intake. These valuable features are key to enhance self-efficacy and self-management with a proper interaction with the case manager in order to monitor progression and subsequently re-adjust the goals periodically (i.e. weekly). Moreover, it is important to highlight that, in terms of the community-based setting, we consider not only traditional indoor physical exercise training sessions, but also outdoor low-tech physical activities allowing exercising at high intensity, such as Nordic walking (48, 49). As such, self-administered community-based high intensity training, with the remote follow-up of a physical therapist, is a plausible option to enhance service delivery.

### Behavioral Change Techniques and Digital Health

It is well known that aerobic capacity is not a determinant factor related with physical activity levels. In this regard, core components to be included in successful behavioral interventions (i.e. physical activity and nutrition) have been reported in several meta-analyses and guidelines (50–55). Therefore, to design effective digital solution to foster an active lifestyle, it is key to implement well-established behavior change techniques for enhancement of complex behaviors, such as physical activity. Most commonly used behavioral change techniques that appeared effective in eHealth interventions in highly prevalent chronic conditions such as cardiovascular conditions, type 2 diabetes, obesity and chronic pain, among others, are already reported (56–58).

On this basis, it is highly recommendable that mobile apps designed to support community-based physical activity and physical exercise training under the umbrella of multimodal prehabilitation programs include the following functionalities: i) information on health consequences of enhancing physical activity levels by means of personalized education information, likely in a video format; ii) personalized instructions on how to perform physical activity, likely in a video format; iii) weekly goal setting; iv) tools for self-monitoring of physical activity level and intensity, heart rate, and symptoms during its practice; and, v) feedback on performance both, automatic, based on predefined rules and goals, and also by means of direct chat with the physical therapist. Moreover, this approach can be also applied in other modules of the prehabilitation program such as nutritional optimization, psychological management, and/or smoking and alcohol cessation.

## Conclusions

Digital innovation is a cornerstone aspect to consider to successfully enable large scale adoption of community-based prehabilitation with the final aim of enhancing access and adherence to these programs. Technological developments should support collaborative work and engagement between stakeholders by customizable and interoperable tools to avoid fragmentation of care. Moreover, it is key to design eHealth solutions for patients including effective behavioral change techniques in order to optimize clinical outcomes. Finally, the digital approach described in the current manuscript have the potential to be adopted at a population level by long-term self-management and healthy lifestyles promotion programs to enhance medical prognosis for most prevalent chronic conditions and for prevention of multimorbidity in subjects at risk.

## Data Availability Statement

The original contributions presented in the study are included in the supplementary material. Further inquiries can be directed to the corresponding authors.

## Author Contributions

All authors contributed to the article and approved the submitted version.

## Funding

Sources of support: PAPRIKA EIT-Health-IDB-19365; FIS-Smart PITeS (PI18/00841); FIS (PI17/00852); Catalan Foundation of Respiratory Medicine (FUCAP 2019); Spanish Respiratory Society (SEPAR 2018; Project 791).

## Conflict of Interest

The authors declare that the research was conducted in the absence of any commercial or financial relationships that could be construed as a potential conflict of interest.
